# Family support modifies the effect of changes to same-sex marriage legislation on LGB mental health: evidence from a UK cohort study

**DOI:** 10.1093/eurpub/ckab139

**Published:** 2021-08-27

**Authors:** Celine Teo, Nicholas Metheny, Antony Chum

**Affiliations:** 1 Department of Applied Health Sciences, Brock University, St Catharines, Ontario, Canada; 2 School of Nursing and Health Studies, University of Miami, Coral Gables, Florida, USA; 3 Epidemiology Division, Dalla Lana School of Public Health, University of Toronto, Toronto, Ontario, Canada; 4 MAP Centre for Urban Health Solutions, Li Ka Shing Knowledge Institute, Unity Health Toronto, Toronto, Ontario, Canada

## Abstract

**Background:**

Many lesbian, gay and bisexual (LGB) individuals continue to experience unique challenges, such as the lack of family support and access to same-sex marriage. This study examines the effect of the introduction of same-sex marriage in the UK (2013–14) on mental health functioning among sexual minorities, and investigates whether low family support may hamper the positive effects of marriage equality legislation among LGB individuals.

**Methods:**

This analysis included LGB participants (*n* = 2172) from the UK household longitudinal study waves 3–7, comprising two waves before and two waves after marriage equality legislation passed in England, Wales and Scotland. Individual-level mental health functioning was measured using the mental component score (MCS-12) of the Short Form-12 survey. Fixed-effect panel linear models examined the effect of marriage equality on MCS-12 across varying family support levels. Analyses included adjustment for covariates and survey weights.

**Results:**

Legalization of same-sex marriage was independently associated with an increase of 1.17 [95% confidence interval (CI): 0.28–2.05] MCS-12 in men and 1.13 (95% CI: 0.47–2.27) MCS-12 in women. For men, each additional standard deviation of family support modified the effect of legalization on mental health functioning by +0.70 (95% CI: 0.22–1.18) MCS-12 score. No interaction was found in women.

**Conclusions:**

Our findings provide evidence that same-sex marriage will likely improve LGB mental health functioning, and these effects may be generalizable to other European countries. Since male sexual minorities with low family support benefited the least, additional interventions aimed at improving family support and acceptance of this group is required to help reduce mental health disparities.

## Introduction

Same-sex marriage legalization is associated with direct benefits for lesbian, gay and bisexual (LGB) people (e.g. spousal health care benefits, tax benefits, adoption, etc.).^1^^,^^2^ Same-sex marriage also presents an indirect social inclusionary effect for all members of the LGB community by normalizing same-sex relationships and increasing sense of belonging in society.^3^ The legalization of same-sex marriage has been linked to a reduction in the proportion of suicide attempts in LGB high school students,^4^ reductions in mental health care costs in sexual minority men,^5^ improved general mental health and subjective well-being,^6^ lowered rates of mental health disorders,^6^^,^^7^ reduced feelings of internalized homophobia and stress^8^^,^^9^ and an improved sense of societal recognition for LGB individuals in relationships.^10^

A growing number of studies demonstrate a positive impact of same-sex marriage on the mental health of LGB individuals.^11–16^ While the current body of quantitative evidence is largely the US focused,^4–9^^,^^16^ several European studies should be mentioned. In a Swedish population-based study from 2005 to 15 (*N* = 23 248 individuals, 565 sexual minorities),^17^ researchers found that the legalization of same-sex marriage (in 2009) led to reductions in structural stigma towards LGB individuals over the period (*r* = –0.90, *P* < 0.001), and in turn, closed the disparity in psychological distress between sexual minority vs. heterosexuals by 2015. In Portugal, following legislative changes allowing same-sex marriage in 2010 and allowing same-sex couples to adopt and jointly adopt children in 2016, interviews with 425 LGB individuals revealed that such policy changes has helped improve social inclusion for the LGB community.^18^

In the UK, the Civil Partnership Act was passed in November 2004, legalizing civil partnerships. Legislations to allow same-sex marriage in England and Wales were passed in July 2013, followed by Scotland in February 2014 and Northern Ireland in July 2019.^19^ While the recognition of same-sex civil partnership was an important milestone for LGB equality, many within the LGB community considered it to be a second-class substitute and denied social legitimacy to same-sex relationships.^3^ Same-sex couples who were legally married reported lower levels of psychological distress and increased well-being compared with those in civil unions.^8^^,^^19^^,^^20^ These findings highlight important differences between civil partnership and marriage, and the unique mental health benefits of same-sex marriage above and beyond legal recognition through civil partnerships.

Despite policy changes increasing the legal inclusion of LGB people in much of Europe since the turn of the century, significant numbers of LGB individuals continue to face unique challenges, such as family rejection and lack of family support that leads to feelings of social exclusion.^21^ Social exclusion, or the inability to fully participate in society due to social, economic and psychological isolation,^22^ is a driving force for health inequalities in sexual minority populations. Social exclusion is linked to negative health outcomes for LGB people due to the increased allostatic load, emotion dysregulation and cognitive processes that confer risk of poor mental health outcomes for individuals.^23–28^ Specifically, Hatzenbuehler’s Integrative Psychological Mediation framework stipulates that discrimination and social exclusion leads to increased proximal stressors in the form of internalized stigma, expectations of rejection, poor self-image and sexual minority identity concealment.^29^ Social support is also an important social determinant of mental health and self-acceptance.^24^^,^^30^ In a longitudinal study examining the influence of various sources of social support (including family, friends and partner support), only family support was uniquely associated with LGB mental health 2 years after social support was first measured,^31^ corresponding to an improvement of 0.17 points on their General Health Questionnaire (GHQ) score for each point in family support (*P* = 0.003). Additional research shows that the lack of LGB family support has been linked to higher rates of mood disorders,^32^^,^^33^ lower levels of psychological well-being,^8^ increased risks of suicide-related behaviours,^32^^,^^34^ more likely to report internalized homophobia^35^ and increased risks of homelessness.^36^ In a study of 224 LGB individuals in the USA, those who reported family rejection and lack of family support had increased odds of suicide attempts by 3.09 times [95% confidence interval (CI): 2.18–4.37], 2.21 times (95% CI: 1.62–3.01) for severe depression (Centre for Epidemiological Studies Depression Score > 16), 1.83 times (95% CI: 1.35–2.49) for using illicit substances and 1.73 times (95% CI: 1.25–2.40) for risky sexual behaviours.^32^ Given the growing evidence of the importance of family support, it is alarming that a lack of family support is a widespread problem among LGB individuals: in a study of 754 LGBT respondents across 37 Western European countries, over half (51%) of the respondents reported experiencing lack of support and/or discrimination from their family members.^21^

While studies show that same-sex marriage has positive social inclusionary effects for LGB individuals,^4–9^^,^^16–18^ there are two main gaps in the literature that this study aims to fill. First, there are few studies that have quantitatively investigated the effect of same-sex marriage legislation on LGB mental health in the UK, and second, whether these effects are homogeneous across LGB individuals with different levels of family support and acceptance. Returning to the Integrative Psychological Mediation framework,^29^ Hatenbuehler draws on Meyer’s Minority Stress theory to develop his model of how minority stressors lead to poor mental health. In his original model, Meyer posits that social support, especially family support, is a key moderator of the relationship between minority stress and health outcomes. Family support may also be a potential effect modifier since (i) the symbolic value of marriage as a signifier for community and familial recognition may be more relevant to individuals who already have high levels of family support and acceptance^37^^,^^38^ and (ii) there may be a chance that the social recognition conferred by marriage equality has social inclusionary effects beneficial to mental health that partially compensate for social exclusion experienced at the family level. Considering the preponderance of evidence supporting the potentially modifying effect of family support between social exclusion and mental health, we hypothesize that an LGB individual’s relationship with their family may modify the effect of marriage equality on their mental health.

The purpose of this study is therefore to examine the effect of the introduction of same-sex marriage in England, Wales and Scotland (2013–14) on mental health functioning among sexual minorities, and to investigate whether family support moderates this relationship. A better understanding of how family support modifies this relationship can highlight points of intervention and potential sub-populations that can be targeted for tailored interventions in the UK and throughout Europe.

## Methods

The study cohort included 2172 LGB individuals drawn from the UK longitudinal household survey (UKHLS),^39^ which is a population-based household survey representative of the UK population. To focus in on the mental health changes that occurred with the introduction of same-sex marriage (occurred during wave 5), our analyses were limited to waves 3–7 of the UKHLS covering the years 2011–17. Respondents aged 16 years and over were included in our analyses. Further information on recruitment strategies, locations, relevant dates, recruitment and follow-up strategies can be found at https://www.understandingsociety.ac.uk/.

To study the impact of the introduction of same-sex marriage, we used a pre–post-study design to examine the change in mental health functioning among LGB individuals, while testing for potential interaction between family support and the introduction of same-sex marriage. Since marriage equality was passed in July 2013 for England and Wales, and February 2014 in Scotland, it occurred during the data collection of wave 5 (2013–15). To determine whether the participants’ responses should be categorized as pre- vs. post-legalization, we compared each participant’s date of interview to the date of legalization in their country of residence at the time of their interview.

Respondents’ sexual orientation was captured on the UKHLS through a single-item question: ‘select one of the following options that best describes your sexuality’. Answers included (i) heterosexual, (ii) homosexual, (iii) bisexual, (iv) others and (v) prefer not to say. Those indicating homosexual, bisexual or others were included in the analysis, and ‘prefer not to say’ (*n* = 1551) were excluded from analysis.

Family support was measured in waves 2 and 5 using six questions on the UKHLS. Participant’s relationship with their family was rated including ‘understanding the way you feel’, ‘can you rely on them when you have a serious problem’, ‘can you open up to them’, ‘how much do they criticise you’, ‘do they let you down’ and ‘do they get on your nerves’ were rated on a four-point scale from ‘a lot’ to ‘not at all’. Scores were summed for each participant and standardized. A prior study found that the instrument has high internal consistency (α = 0.84) and had predictive validity for general mental health and psychiatric distress as measured by GHQ-12. We aggregated family support scores across both waves for a more stable estimate of long-term family support. This is supported by previous studies where family support exhibited minimal variability in an individual’s life course.^40–42^

### Study outcome

The mental component score (MCS-12) of the Short Form-12 survey is a validated tool to measure mental functioning and has been used as a screening tool for depression and anxiety disorders.^43^ MCS-12 measures the respondent’s mental health state in the last four weeks, and is calculated based on norm-based scoring (with higher scores indicating better mental health), which linearly transforms the scales and summary measures to have a mean of 50 and SD of 10 based on English general population data.^44^ Previous study finds that the MCS-12 is highly associated with the Colorado Symptoms Index (*r* = –0.650, *P* < 0.001) for psychiatric symptomatology.^45^

### Control variables

Our statistical method utilizes a within-subject design, which automatically controls for all observed and unobserved time-invariant characteristics. By modelling only the within-person change in mental health over time, the model effectively controls for the effects of time-invariant factors (e.g. ethnicity) since each person acts as their own control.^46^ Therefore, we restrict our identification of potential confounders to those that vary over time, which include: (i) friend social support (measured using the same questions as family support—replacing the word family with friends), (ii) household income in the last month (£), (iii) adverse health condition (presence of a diagnosed mental or physical chronic condition in the last 12 months), (iv) natural log of age, (v) LGB density in the participant’s region of residence (derived through the Annual Population Survey from 2012 to 18)^47^ and (vi) residential relocation since the last wave. Since there may be factors that could simultaneously affect individuals across the UK (e.g. recessions, Brexit, etc.), a year fixed-effect was included to account for trend effects and temporal changes in mental health over time. Importantly, this also controls for changes in the broader social acceptance of LGB people over time. A household-level fixed-effect was used to account for unique unobserved household characteristics such as poor or unsafe living conditions. A region-level fixed-effect (11 regions) was applied to control for persistent regional differences, including the differing rates of LGB social acceptance across the UK.

### Statistical analysis

A fixed-effects, within-person, regression analysis was applied to this study to investigate the impact of legalizing same-sex marriage on LGB mental health functioning, and to determine if the effect of legalization varied by an individual’s degree of family support. We fit three models total. Model 1, with person-wave observations as the unit of analysis, regressed MCS-12 on: (i) pre- vs. post-legalization indicator, (ii) individual, year, household and region fixed-effects, (iii) time-variant control variables as mentioned above and (iv) interaction between the legalization indicator and family support. Models 2 and 3 stratified the sample by gender, Model 2 is based on male-only subsample and Model 3 is female-only. For all three models, we also tested for potential interactions between legalization and (i) sexual orientation (i.e. homosexual, bisexual and others) and (ii) age. Survey weighting and multiple imputation of missing variables were used in this analysis to minimize potential bias from selection of participants and non-responses. All models were estimated using the PLM package 2.2-3 in R Studio.

## Results

**Figure 1:**
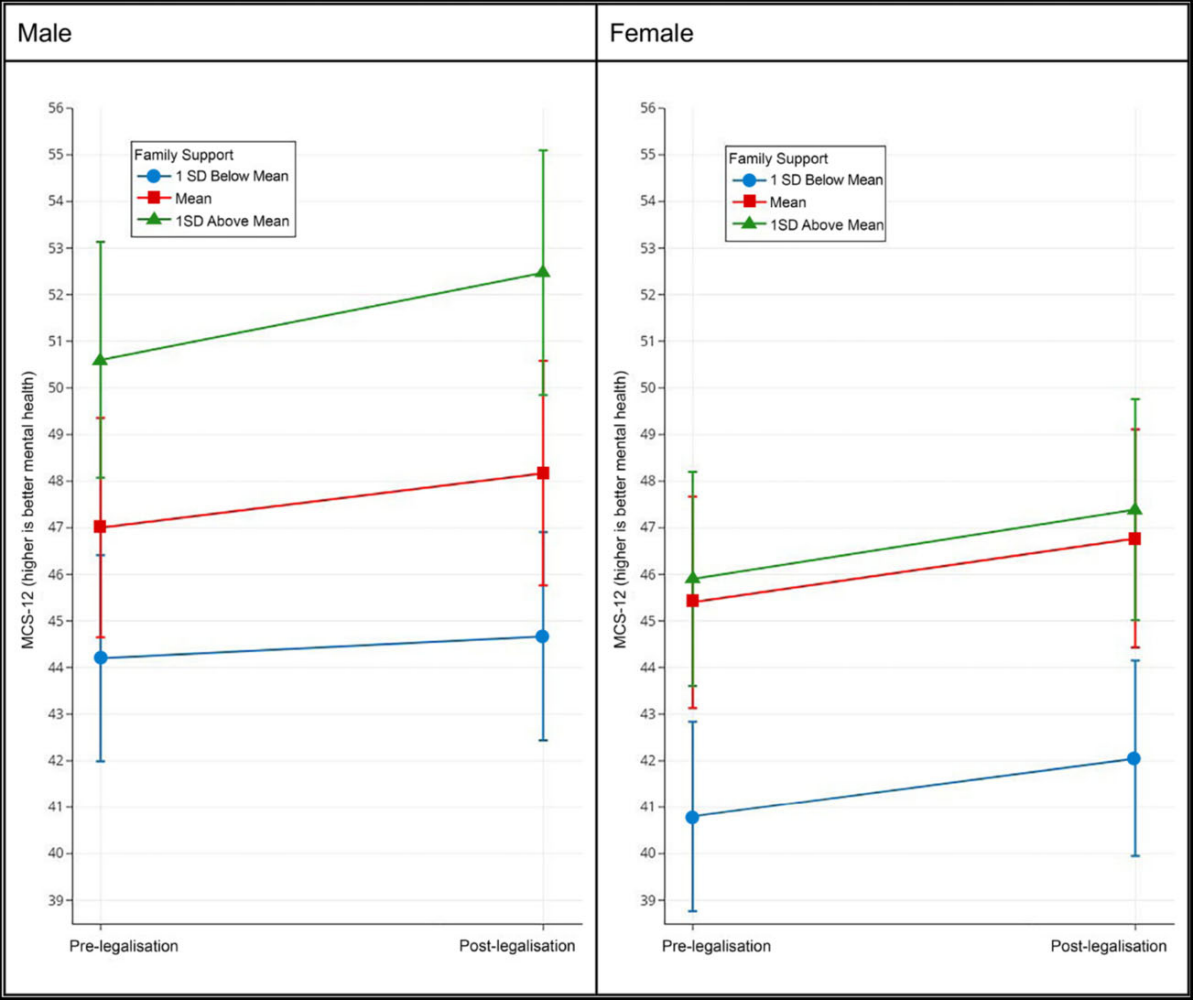
Plot of family support as an effect modifier of same-sex marriage legalization for men vs. women

Table 1 presents the sample distribution stratified by gender at baseline along with the gender-stratified mean MCS-12 scores at each level of the exposure. Based on the table, bisexual women, younger participants, those with low family and friend support, individuals with low household income, presence of an adverse health condition and not married individuals appear to have a lower MCS-12 score. While we considered responses from waves 3–7 of the UKHLS (five waves), the average number of survey responses were 4.29 waves (SD = 1.11) across study participants. We also found evidence that the legalization of same-sex marriage was independently associated with an improvement in LGB mental health functioning in the full model (B = 1.26, 95% CI: 0.62–1.89), *P* < 0.001, as well as in both sex-stratified models (Model 2: B = 1.17, 95% CI: 0.28–2.05, *P* < 0.01; Model 3: B = 1.37, 95% CI: 0.47–2.27, *P* < 0.01). Each increase of 1000 pounds Sterling in household income was associated with one-third of a point increase in MCS-12 score for women (B = 0.34, 95% CI: 0.07–0.62, *P* < 0.05), but not for men or in the full model. In all three models, having an adverse health condition was associated with a statistically significant increase in MCS-12 score (Model 1: B = 1.29, 95% CI: 0.60–1.97, *P* < 0.001; Model 2: B = 0.50, 95% CI: 0.11–2.11, *P* < 0.05; Model 3: B = 1.44, 95% CI: 0.50–2.37, *P* < 0.01). Age, viewed logarithmically, was negatively associated with MCS-12 score in all three models, though less so for women than men (Model 1: B+ –15.1, 95% CI: –21.7 to –8.56, *P* < 0.001; Model 2: –18.0, 95% CI: –27.2 to –8.78, *P* < 0.001; Model 3: B = –12.2, 95% CI: –22.6 to –3.91, *P* < 0.001). Moving residences since the last wave was also significantly associated with a lower score on the MCS-12 for the full model (B = –2.14, 95% CI: –3.39 to –0.088, *P* < 0.001) and for women (B = –2.52, 95% CI: –4.25 to –0.79, *P* < 0.01). The effect of legalization on mental health functioning was higher for men with higher degrees of family support (compared with men with low family support), but family support did not modify the effect of legalization for women (see table 2 and figure 1).

**Table 1:** Baseline characteristics, *n* = 993 for men and *n* = 1179 for women

Sample characteristics		Proportions for males (*n*)	Proportions for females (*n*)	Mean MCS-12 (*SD*)—male	Mean MCS-12 (*SD*)—female
Sexuality	Homosexual	43.0% (427)	24.8% (292)	45.9 (10.9)	46.0 (10.7)
	Bisexual	31.4% (312)	43.6% (514)	46.4 (10.3)	41.8 (12.6)
	Others	25.6% (254)	31.6% (373)	47.8 (10.6)	45.3 (11.5)
	Missing	0	0	–	–
Age	<25 years	36.3% (361)	40.0% (472)	45.6 (10.7)	41.7 (12.6)
	26–45 years	30.3% (301)	32.9% (388)	46.3 (10.8)	44.2 (11.3)
	46–64 years	24.5% (243)	19.0% (224)	46.4 (10.9)	46.1 (11.2)
	65+ years	8.9% (88)	8.1% (95)	51.4 (7.74)	48.2 (10.7)
	Missing	0	0	–	–
Family support	Low (<0.66)	25.7% (255)	24.4% (288)	44.2 (11.0)	40.9 (12.4)
	Medium (0.66–0.81)	27.8% (276)	24.4% (288)	46.7 (10.4)	45.6 (10.4)
	High (>0.81)	26.6% (264)	31.0% (365)	48.5 (10.2)	46.5 (11.2)
	Missing	19.9% (198)	20.2% (238)	46.9 (10.5)	41.1 (13.1)
Friend support	Low (<0.72)	28.7% (285)	22.9% (270)	44.6 (11.5)	41.2 (12.6)
	Medium (0.72 – 0.83)	23.6% (234)	20.5% (242)	47.2 (9.71)	45.2 (11.2)
	High (>0.83)	27.9% (277)	36.6% (431)	47.9 (10.3)	46.2 (10.8)
	Missing	19.8% (197)	20.0% (236)	46.8 (10.5)	41.1 (13.1)
Household income	Quartile 1 (lowest)	24.5% (243)	25.4% (300)	44.8 (11.5)	42.8 (12.5)
	<£1628.74				
	Quartile 2	26.5% (263)	23.8% (280)	45.8 (10.6)	43.9 (12.2)
	£1628.74–2491.41				
	Quartile 3	24.0% (239)	25.8% (304)	48.5 (10.3)	43.6 (11.7)
	£2491.41–3682.74				
	Quartile 4 (highest)	25.0% (248)	25.0% (295)	47.2 (9.66)	45.4 (11.2)
	> £3682.74				
	Missing	0	0	–	–
Presence of adverse health condition	Yes	30.4% (302)	31.7% (374)	43.4 (12.0)	40.4 (12.5)
	No	69.2% (687)	68.3% (805)	48.0 (9.65)	45.7 (11.2)
	Missing	0.4% (4)	0	38.4 (11.9)	–
Marital status	Single + divorced + widowed	70.5% (700)	67.6% (797)	45.7 (11.0)	42.8 (12.3)
	Married + civil partner	27.0% (268)	29.8% (351)	49.0 (9.27)	46.5 (10.8)
	Missing	2.5% (25)	2.2% (31)	*52.4* (NA)^a^	NA^a^

a NA, Not Applicable; out of all participants that had missing marital status, only one participant at baseline had a valid answer for MCS-12.

**Table 2:** Fixed-effect (within-subject) regressions predicting change in MCS-12 associated with the legalization of same-sex marriage

Predictors	Model 1 (combined for men and women, *n* = 1728)	Model 2 (men only, *n* = 792)	Model 3 (women only, *n* = 936)
	Estimates (95% CI)	Estimates (95% CI)	Estimates (95% CI)
Legalization of same-sex marriage	1.26 (0.62–1.89)^***^	1.17 (0.28–2.05)^**^	1.37 (0.47–2.27)^**^
Household Income (in £1000)	0.06 (–0.02 to 0.14)	0.02 (–0.05 to 0.11)	0.34 (0.07–0.62)^*^
Adverse health condition	1.29 (0.60–1.97)^***^	0.50 (0.11–2.11)^*^	1.44 (0.50–2.37)^**^
Marital status (0 for not married, 1 for married)	0.60 (–0.71 to 1.91)	1.19 (–0.84 to 3.23)	0.12 (–1.61 to 1.87)
Age (natural log)	–15.1 (–21.7 to –8.56)^***^	–18.0 (–27.2 to –8.78)^***^	–12.2 (–22.6 to –3.91)^**^
LGB regional density (% of population identifying as LGB)	0.02 (–1.11 to 1.17)	–0.18 (–1.75 to 1.38)	0.19 (–1.44 to 1.83)
Residential relocation	–2.14 (–3.39 to –0.88)^***^	–1.21 (–3.08 to 0.64)	–2.52 (–4.25 to –0.79)^**^
Legalization of same-sex marriage × family support (z-score)	0.43 (0.06–0.79)^*^	0.70 (0.22–1.18)^**^	0.12 (–0.43 to 0.67)

Note: All models above additionally controlled for individual-level fixed-effect, year fixed-effect, region fixed-effect and household fixed-effect.

* *P *< 0.05.

** *P *< 0.01.

*** *P *< 0.001.

In Model 1 (male and female), we found evidence that each standard deviation increase in family support modified the effect of legalization on mental health by 0.43 (95% CI: 0.06–0.79) points on the MCS-12 score. For the male-only model (Model 2), we found that for each standard deviation increase in family support modified the effect of legalization on mental health functioning by 0.70 (95% CI; 0.22–1.18) MCS-12 score. In other words, individuals who are 1 SD below the mean family support are estimated to have an improvement of 0.47 points on the MCS-12 (95% CI: 0.06–0.87), while individuals who are 1 SD above the mean family support are estimated to have an improvement of 1.87 (95% CI: 0.50–3.23). See figure 1 for visualization of the interaction effect in men vs. the lack of effect modification in women. In separate analyses, we did not find evidence of effect modification of legalization based on age or sexual orientation.

## Discussion

Our study adds to the growing evidence that the legalization of same-sex marriage has an independent positive effect on mental health of LGB individuals.^11–18^ While the effect size for the direct effect of legalization is modest (i.e. an estimated improvement of 1.26 MCS-12 score in light of an SD of 10 in the English general population),^44^ this coefficient should be understood in the context of a tightly controlled within-subject model. Within-subject models, as used in our analyses, are known to produce more conservative estimates (with a downward bias) compared with random-effect models^48^ since they only model the portion of variance in the outcome that changes within-person over time and ignores between-person variance. In addition, while an improvement of 1.26 MCS-12 may be modest at the individual level, a systemic improvement of the same amount across the UK LGB subpopulation (that have historically suffered from disproportionately higher levels of mental health disorders^9^^,^^10^) would be a significant measurable outcome if legalization of same-sex marriage is considered as a national public health intervention.

Our study provides evidence that family support continues to be an important determinant of mental health for sexual minorities, and can influence LGB mental health indirectly by modifying the effect of marriage equality policies. Mechanistically, the results may follow the expected pathways laid out by the psychological mediation framework.^29^ The legalization of same-sex marriage is shown in this study to be beneficial in its own right, potentially by reducing distal minority stressors via improving social inclusion. However, given the importance of family support in buffering the transformation of distal stressors into proximal stressors such as identity concealment and internalized homophobia,^49^,^50^ it stands to reason that those with higher levels of these types of minority stress are unable to realize the full benefits equal marriage brings to the social inclusion of LGB people, especially gay and bisexual men. This notion is corroborated by previous studies of marriage equality and minority stress in the USA.^51^,^52^ This points to the need for policies and interventions that work both at the macro-level (i.e. marriage legalization) as well as at the community and household levels to increase support for LGB individuals, and may be tailored to gay and bisexual men. Interestingly, family support did not moderate the relationship between marriage equality and MCS-12 score in Model 3 (women only), though the directionality was the same as in Models 1 and 2. This may be because Western societies have traditionally held less negative attitudes towards same-sex sexuality in women than in men. This may lead to lower levels of distal, and therefore proximal, minority stressors and therefore an attenuated effect of family support. However, lesbian and bisexual women also sit at the intersection of two marginalized identities—being female in a patriarchal society and sexual minority status, potentially decreasing the valence of their sexual identity to their overall sense of self when compared with gay or bisexual men. This could mean that female sexual minorities have an increased (but diversified) discrimination, which may also result in an attenuated effect of family support on the relationship between marriage equality and mental health functioning.

There was no evidence from our study that the effects of legalizing same-sex marriage on mental health differed across age or within sexual minorities groups. It is possible that we were unable to find any effect modification due to small cell sizes in some categories, which could result in Type-II error. Future studies that are sufficiently powered to investigate effect modification by age and sexual minority groups are required to rule out the possibility of effect heterogeneity of marriage equality across these categories.

There are a number of limitations of this study: (i) The UKHLS survey did not include certain questions on gender identity (i.e. transgender and non-binary), limiting the scope of our study to sexual minorities. It should be noted that the participants are free to change their gender response (male vs. female) across waves. (ii) The study’s measure of family support was only collected in the pre-legalization period (waves 2 and 5) since these questions were not available in the post-legalization waves. However, prior research provides evidence that family support (i.e. social and emotional support) are relatively time-stable as they reflect durable family environments and parental styles. Based on individuals aged 20–64 years in the National Survey of Families and Households (*n* = 7366), researchers found no evidence that the level of family emotional and social support changed significantly over an individual’s life course.^40^ (iii) Information on whether the participant had disclosed their sexual orientation to their family was unavailable and could be considered a potential confounder. (iv) Finally, there may be additional time-variant factors that are associated with improvements in mental health over the course of the study beyond what could be controlled for through the year fixed-effect. Our findings provide evidence that legislation for same-sex marriage improved LGB mental health in the UK context, and these positive public health effects may be generalizable to other European countries. For instance, there are still currently 13 countries in the EU that have not legalized same-sex marriage (e.g. Italy, Greece, etc.). While legalization improved LGB mental health as a whole, these improvements were not evenly distributed, and male sexual minorities with low family support experienced the least improvement in mental health functioning. This points to the need for additional interventions for this group. Programs that help families accept their LGBT children, such as the Family Acceptance Project,^53^ may be promising interventions that can target this vulnerable subgroup. Given the effectiveness of legalizing same-sex marriage in improving LGB mental health functioning, future research should also evaluate other LGB-related social policy changes such as bans on conversion therapy and its impact on the mental health of sexual minorities.

## Supplementary data

Supplementary data are available at *EURPUB* online.

## Funding

Funding for the project is provided through research start-up funds from Brock University, Faculty of Applied Health Science, to the project principal investigator, Antony Chum. The funder had no role in determining the topic, scope or interpretation of study results.

*Conflicts of interest*: None declared.

**Key points**

- Findings provide evidence that legislation for same-sex marriage improved LGB mental health in the UK context.
- Gay and bisexual men with low family support benefited the least from same-sex marriage legislation.
- European countries that do not recognize same-sex marriage needs to consider the positive public health effects this policy change has on the LGB community.
- Legalization of same-sex marriage may help close mental health disparities across sexual orientations in other European countries where it is not currently legal.

## Supplementary Material

ckab139_Supplementary_Data
