# Supplementary material for: Family support modifies the effect of changes to same-sex marriage legislation on LGB mental health: evidence from a UK cohort study
Source: Eur J Public Health. 2021 Aug 27;32(1):35–40. doi: 10.1093/eurpub/ckab139 (PMC9090167; doi:10.1093/eurpub/ckab139)
Supplement: ckab139_Supplementary_Data [file ckab139_supplementary_data.docx]

**Supplementary File**

**References (cont.)**

41. Bucx F, Wel F van, Knijn T. Life Course Status and Exchanges of Support Between Young Adults and Parents. J Marriage Fam. 2012;74(1):101–15.

42. Reczek C. The Intergenerational Relationships of Gay Men and Lesbian Women. J Gerontol Ser B. 2014 Nov 1;69(6):909–19.

43. Gill SC, Butterworth P, Rodgers B, Mackinnon A. Validity of the mental health component scale of the 12-item Short-Form Health Survey (MCS-12) as measure of common mental disorders in the general population. Psychiatry Res. 2007 Jul 30;152(1):63–71.

44. Layte R, Jenkinson C. Normative Data for the SF-12 Health Survey In the Republic of Ireland with Comparisons to England. Ir J Psychol. 2001 Jan;22(1):63–72.

45. Chum A, Skosireva A, Tobon J, Hwang S. Construct Validity of the SF-12v2 for the Homeless Population with Mental Illness: An Instrument to Measure Self-Reported Mental and Physical Health. Seedat S, editor. PLOS ONE. 2016 Mar 3;11(3):e0148856.

46. Gunasekara FI, Richardson K, Carter K, Blakely T. Fixed effects analysis of repeated measures data. Int J Epidemiol. 2014 Feb;43(1):264–9.

47. Sexual orientation, UK - Office for National Statistics [Internet]. [cited 2020 Aug 6]. Available from: https://www.ons.gov.uk/peoplepopulationandcommunity/culturalidentity/sexuality/datasets/sexualidentityuk

48. Hill TD, Davis AP, Roos JM, French MT. Limitations of Fixed-Effects Models for Panel Data. Sociol Perspect. 2020 Jun 1;63(3):357–69.

49. Feinstein BA, Wadsworth LP, Davila J, Goldfried MR. Do parental acceptance and family support moderate associations between dimensions of minority stress and depressive symptoms among lesbians and gay men? Prof Psychol Res Pract. 2014;45(4):239–46.

50. Rostosky SS, Riggle EDB, Gray BE, Hatton RL. Minority stress experiences in committed same-sex couple relationships. Prof Psychol Res Pract. 2007;38(4):392–400.

51. Metheny N, Stephenson R. Political Environment and Perceptions of Social Inclusion After Nationwide Marriage Equality Among Partnered Men Who Have Sex with Men in the USA. Sex Res Soc Policy. 2019 Dec;16(4):521–8.

52. Gonzalez KA, Ramirez JL, Galupo MP. Increase in GLBTQ minority stress following the 2016 US presidential election. J GLBT Fam Stud. 2018;14(1–2):130–51.

53. Ryan C. Engaging Families to Support Lesbian, Gay, Bisexual, and Transgender Youth: The Family Acceptance Project: (509042011-003) [Internet]. American Psychological Association; 2010 [cited 2020 Jul 30]. Available from: http://doi.apa.org/get-pe-doi.cfm?doi=10.1037/e509042011-003
